# Reply to Emery-Wetherell: Taphonomy of Lujiatun 3D dinosaurs is inconsistent with death and burial by lahars

**DOI:** 10.1073/pnas.2423567122

**Published:** 2025-01-09

**Authors:** Paul E. Olsen, Scott A. MacLennan, Jingeng Sha, Sean T. Kinney, Clara Chang, Yanan Fang, Jun Liu, Bennett B. Slibeck, Elaine Chen, Blair Schoene

**Affiliations:** ^a^Biology and Paleo Environment, Lamont-Doherty Earth Observatory of Columbia University, Palisades, NY 10964; ^b^School of Geosciences, University of the Witwatersrand, Johannesburg 2017, South Africa; ^c^Department of Geosciences, Princeton University, Princeton, NJ 08544; ^d^State Key Laboratory of Palaeobiology and Stratigraphy, Nanjing Institute of Geology and Palaeontology, Nanjing 210008, China; ^e^Earth and Planetary Sciences Rutgers University, Piscataway, NJ 08854; ^f^Department of Paleoichthyology and Paleoherpetology, Institute of Vertebrate Paleontology and Paleoanthropology, Chinese Academy of Sciences, Beijing 100044, China; ^g^Private address, New York, NY 10019

We welcome Emery-Wetherell’s (1) comments on our study of the Early Cretaceous Yixian Formation in northeast China (2). Detailed attention to taphonomy of this assemblage, especially the three-dimensional (3D) preservation of dinosaurs and mammals in the Lujiatun Member, is overdue. However, Emery-Wetherell raises several issues that warrant further discussion.

Although we agree that grain-size gradients are not exclusive to burrow collapses, they do require rapid burial of nonskeletonized animals, which is inconsistent with significant delays between death and burial. Additionally, the positions of the Lujiatun skeletons do not support transport by currents, mudflows, or lahars, as these would cause disorientation and movement, which we do not observe in the complete skeletons. The “full-body imbrication” and “limb entanglement” cited as evidence of transport inconsistent with burrow collapse (1) actually align extremely well with normal juvenile huddling (3) or combat (4), respectively, within burrows.

Although it is true that lahars may pick up nonvolcanic material as they travel, that in no way excludes other mechanisms of mixing such as fluvial redeposition or pedogenic processes. Moreover, the dinosaur-bearing Lujiatun units we studied lack large volcanic clasts typical of lahars and differ from modern lahar examples described by Smith and Lowe (5). It is nonetheless plausible that some Lujiatun beds are lahars (e.g., ref. 6). It is also possible that dinosaurs and other animals dug into previously deposited lahars and were thereby preserved in burrows but neither means that the dinosaurs or mammals were preserved by the lahar emplacements themselves.

We contest that the absence of crushing or compression evidence rules out burrow entombment. The skeletons were buried under many meters of sediment and lava and subjected to compaction regardless of their initial burial mechanisms. Furthermore, some Lujiatun specimens do exhibit significant compaction despite being fully articulated (e.g., ref. 7, figure 2), and the factors influencing compaction levels remain unexplored.

Emery-Wetherell (1) suggests that our arguments (2) are undermined by the fact that many burrowing animals dig their way out after burrow collapse. We disagree; animals that escape would not be preserved in burrows and thus their survival is not pertinent to this discussion. Furthermore, even in the referenced penguin study (8), adult mortality from burrow collapse is possible during a “total burrow cave-in” and is a cause of mortality as noted by Stokes and Boersma (9).

We concur that CO_2_ poisoning could have killed some Lujiatun animals but see no reason it could not have occurred within burrows. However, the notion that most Lake Nyos livestock and human victims died in sleeping positions lacks published photographic support (e.g., ref. 10), although survivor accounts indicate some people did indeed die this way (e.g., ref. 11). However, this does not clarify how so many Lujiatun animals were buried in sleeping positions.

Unfortunately, crucial information about the sedimentary context of Lujiatun 3D skeletons was lost during excavation by collectors [e.g., “farmers” (7), figure 2]. Our hypotheses about burrow collapse clearly need future testing through analysis of in situ skeletons and modern burrow taphonomy studies.
